# *Plasmodium malariae* and *Plasmodium ovale* infections in the China–Myanmar border area

**DOI:** 10.1186/s12936-016-1605-y

**Published:** 2016-11-15

**Authors:** Peipei Li, Zhenjun Zhao, Hua Xing, Wenli Li, Xiaotong Zhu, Yaming Cao, Zhaoqing Yang, Jetsumon Sattabongkot, Guiyun Yan, Qi Fan, Liwang Cui

**Affiliations:** 1 Dalian Institute of Biotechnology, Dalian, Liaoning China; 2 Dalian University of Technology, Dalian, Liaoning China; 3 Department of Immunology, China Medical University, Shenyang, Liaoning China; 4 Department of Parasitology, Kunming Medical University, Kunming, Yunnan China; 5 Faculty of Tropical Medicine, Mahidol University, Bangkok, 10400 Thailand; 6 University of California, Irvine, CA USA; 7 Department of Entomology, Pennsylvania State University, University Park, PA USA

**Keywords:** *Plasmodium falciparum*, *Plasmodium vivax*, *Plasmodium malariae*, *Plasmodium ovale*, Prevalence, Genetic diversity, Molecular identification

## Abstract

**Background:**

The Greater Mekong Subregion is aiming to achieve regional malaria elimination by 2030. Though a shift in malaria parasite species predominance by *Plasmodium vivax* has been recently documented, the transmission of the two minor *Plasmodium* species, *Plasmodium malariae* and *Plasmodium ovale* spp., is poorly characterized in the region. This study aims to determine the prevalence of these minor species in the China–Myanmar border area and their genetic diversity.

**Methods:**

Epidemiology study was conducted during passive case detection in hospitals and clinics in Myanmar and four counties in China along the China–Myanmar border. Cross-sectional surveys were conducted in villages and camps for internally displaced persons to determine the prevalence of malaria infections. Malaria infections were diagnosed initially by microscopy and later in the laboratory using nested PCR for the SSU rRNA genes. *Plasmodium malariae* and *P. ovale* infections were confirmed by sequencing the PCR products. The *P. ovale* subtypes were determined by sequencing the *Pocytb*, *Pocox1* and *Pog3p* genes. Parasite populations were evaluated by PCR amplification and sequencing of the *MSP*-*1* genes. Antifolate sensitivity was assessed by sequencing the *dhfr*-*ts* and *dhps* genes from the *P. malariae* and *P. ovale* isolates.

**Results:**

Analysis of 2701 blood samples collected from the China–Myanmar border by nested PCR targeting the parasite SSU rRNA genes identified 561 malaria cases, including 161 *Plasmodium falciparum*, 327 *P. vivax*, 66 *P. falciparum*/*P. vivax* mixed infections, 4 *P. malariae* and 3 *P. ovale* spp. *P. vivax* and *P. falciparum* accounted for >60 and ~30% of all malaria cases, respectively. In comparison, the prevalence of *P. malariae* and *P. ovale* spp. was very low and only made up ~1% of all PCR-positive cases. Nevertheless, these two species were often misidentified as *P. vivax* infections or completely missed by microscopy even among symptomatic patients. Phylogenetic analysis of the SSU rRNA, *Pocytb*, *Pocox1* and *Pog3p* genes confirmed that the three *P. ovale* spp. isolates belonged to the subtype *P. ovale curtisi*. Low-level genetic diversity was detected in the *MSP*-*1*, *dhfr* and *dhps* genes of these minor parasite species, potentially stemming from the low prevalence of these parasites preventing their mixing. Whereas most of the *dhfr* and *dhps* positions equivalent to those conferring antifolate resistance in *P. falciparum* and *P. vivax* were wild type, a new mutation S113C corresponding to the S108 position in *pfdhfr* was identified in two *P. ovale curtisi* isolates.

**Conclusions:**

The four human malaria parasite species all occurred sympatrically at the China–Myanmar border. While *P. vivax* has become the predominant species, the two minor parasite species also occurred at very low prevalence but were often misidentified or missed by conventional microscopy. These minor parasite species displayed low levels of polymorphisms in the *msp*-*1*, *dhfr* and *dhps* genes.

**Electronic supplementary material:**

The online version of this article (doi:10.1186/s12936-016-1605-y) contains supplementary material, which is available to authorized users.

## Background

Malaria in humans is caused by four main *Plasmodium* species (*Plasmodium falciparum*, *Plasmodium vivax*, *Plasmodium malariae* and *Plasmodium ovale*) and the zoonotic parasite *Plasmodium knowlesi*, which is found in many Southeast Asian countries [1]. The most virulent species *P. falciparum* is also the most prevalent parasite in Africa, while *P. vivax* is the most widely distributed parasite outside of Africa [2]. Compared with these two species, *P. malariae* and *P. ovale* spp. are much less prevalent and significantly under-studied. *Plasmodium malariae* is more or less sympatric with *P. falciparum* in distribution, mainly found in sub-Saharan Africa and southwest Pacific [3, 4]. In comparison, *P. ovale* spp. were thought to have a much more limited distribution and mostly found in Africa and some islands of the west Pacific [5]. Most published data showed that the prevalence of these two species has been underestimated apparently due to low parasitaemia, morphological resemblance with *P. vivax*, and occurrence as mixed infections with the major parasite species [6–10]. The advent of PCR-based molecular diagnosis method has revolutionized the detection of low-density *Plasmodium* infections [11]. Molecular genotyping also led to the division of *P. ovale* spp. into two distinct subspecies: *P. ovale curtisi* (*Poc*, the classic type) and *P. ovale wallikeri* (*Pow*, the variant type) [12]. The nuclear genome sequences further confirmed that *Poc* and *Pow* are genetically distinct but morphologically indistinguishable [13]. Further refinements and applications of the molecular methods have enabled enhanced detection of these parasites in molecular surveillance in endemic countries (e.g., [14–17]) as well as imported cases [18].

In the Greater Mekong Subregion (GMS) of Southeast Asia, which includes Cambodia, China, Laos, Myanmar, Thailand, and Vietnam, recent achievements in malaria control have motivated countries within this region to plan for regional malaria elimination by 2030. In this region, all five *Plasmodium* parasites infecting humans co-exist [19], and *P. vivax* and *P. falciparum* are the predominant parasite species. In contrast, other parasite species have been detected at much lower prevalence. *Plasmodium malariae* infections have been described in Cambodia [20–22], China [16, 23], Laos [19], Myanmar [24, 25], Thailand [11, 26–28], and Vietnam [19, 29, 30]. The detection of field isolates with variations in both morphology and the SSU rRNA gene suggests that *P. malariae* may exhibit high genetic diversity [23, 24]. Similarly, *P. ovale* spp. have been found in all nations of the GMS including Cambodia [21, 22], Laos [31, 32], Myanmar [16, 33, 34], Thailand [11, 26, 27], and Vietnam [30, 35]. Although a *P. ovale*-like case was described in 1941 in Yunnan [36], most, if not all, *P. ovale* spp. reported in China in recent years were imported, mainly from Africa [37, 38]. Both the classic and variant types of *P. ovale* spp. were identified in autochthonous as well as imported cases [19, 37].

In the final stage of malaria elimination, new strategies tailored for rapid identification of new cases, prevention of local spread, and efficient management of malaria introduction are critical. Especially, malaria in the GMS is highly heterogeneous with transmission foci located along international borders [39]. Hence, malaria re-introduction as a result of cross-border human migration needs to be dealt with, and malaria elimination may require regional cooperative initiatives [40]. Routine malaria surveillance in endemic regions largely relies on microscopy of Giemsa-stained blood smears and rapid diagnostic tests (RDT). Given that *P. malariae* and *P. ovale* infections often present with a low parasitaemia and occur as mixed infections with *P. falciparum* and *P. vivax* [23, 33], molecular methods of detection provide a more accurate estimate of malaria epidemiology. In this study, blood samples collected from the China–Myanmar border area were analysed for malaria parasites using a nested PCR method.

## Methods

### Study sites

This molecular epidemiology study was carried out in two study sites on both sides of the China–Myanmar border. In Myanmar, passive case detection (PCD) was performed in a hospital located in the Laiza Township and several nearby malaria clinics, Kachin State, between May 2011 and December 2012. In 2012, to obtain malaria prevalence information, cross-sectional surveys (CSS) were conducted in ten surrounding villages. In addition, active case detection (ACD) was also conducted through weekly (April–September) or biweekly (October–March) home visits to assess clinical malaria. From 2010, both ACD and PCD activities were started in three nearby camps for internally displaced people (IDP), which were newly established as a result of internal military strife. The Myanmar study site is located in one of the designated Special Zones [25], where malaria burden is particularly heavy and local public health infrastructure is poor. In China, PCD was performed in county and township hospitals of Tengchong, Yingjiang, Longchuan and Ruili counties, Yunnan Province in 2011–2012. Yunnan is the most malaria prevalent province in China and imported cases as cross-border migration made up a major part of malaria cases in Yunnan in recent years [38, 41].

### Malaria parasite samples

In Kachin, 1106 blood samples were obtained during PCD activities from febrile patients attending the hospital and malaria clinics, and 638 were from participants of two CSS (Table 1). In addition, 854 samples were collected from participants in three nearby IDP camps in 2012. Among them, 366 were from febrile patients attending the malaria clinic, 411 were healthy people during the CSS, and 77 were febrile patients identified during weekly or bi-weekly ACD visits. Further, 103 samples were collected from malaria patients attending hospitals in the four counties of Yunnan in 2011–2012. During these surveys, written informed consent and assent for minors were obtained and demographic data were collected using questionnaires. All diagnosis was done by microscopic examination of both thick and thin blood smears. All samples collected from Yunnan Province were evaluated by RDT (Malaria Pv/Pf Test Device, Tycolpharm Co., Limited, UK), a Pf/Pan device based on pan-pLDH [34]. Finger-prick blood drops were collected by trained nurses on Whatman 3M filter paper, air-dried, and stored at −20 °C before processing. Parasite density was estimated using thick smears assuming 8000 leukocytes/μL of blood [34]. The study protocol was reviewed and approved by the Institutional Review Boards of the Pennsylvania State University, USA, Kunming Medical University, China and the local Bureau of Health, Kachin State, Myanmar.

**Table 1:** Surveillance of malaria in Kachin State, Myanmar and four border counties of Yunnan Province, China

Location	Surveys	Method	Pf	Pv	Pm	Po	Pf and Pv	Total positive	Total
Myanmar	PCD	Microscopy	128	253	1	0	34	416 (28.3%)	1472
		PCR	136	257	3	2	63	461 (31.3%)	
	CSS	Microscopy	4	13	0	0	3	20 (1.9%)	1049
		PCR	8	22	0	0	0	30 (2.9%)	
	ACD	Microscopy	0	1	0	0	0	1 (1.3%)	77
		PCR	0	1	0	0	0	1 (1.3%)	
Yunnan	PCD	RDT	18	42	0	0	1	61 (59.2%)	103
		PCR	17	47	1	1	3	69 (67.0%)	

*Plasmodium* species: Pf (*P. falciparum*), Pv (*P. vivax*), Pm (*P. malariae*), and Po (*P. ovale*)

*PCD* passive case detection; *CSS* cross-sectional surveillance; *ACD* active case detection; *RDT* rapid diagnostic test

### Screening for *Plasmodium malariae* and *Plasmodium ovale* spp

Genomic DNA was extracted from dried blood spots on filter paper using the Qiagen DNA Mini Kit according to the manufacturer’s instruction (Qiagen, Germany). Nested PCR was performed to screen malaria infectious from these 2701 blood samples. Primers rPLU5 and rPLU6 were used for the primary PCR, whereas primers rFAL1/2, Pv18SF/R, Pm18SF/R, Po18SF/R, and Pk18SF/R, specific for each of the four human malaria parasite species as well as *P. knowlesi*, were used for the nested PCR [42]. The PCR positive samples of *P. malariae* and *P. ovale* spp. were further confirmed by sequencing the PCR products. Some of the blood smears with inconsistent diagnosis results between the original microscopy and PCR were re-examined to verify the presence of parasite-infected red blood cells.

### Molecular characterization of *P. malariae* and *P. ovale* isolates

To further characterize the *P. malariae* and *P. ovale* isolates, several genes were amplified and sequenced using primers listed in Additional file 1. These include the *cytochrome b* (*cytb*), *cytochrome oxidase subunit 1* (*cox1*) and *glyceraldehyde*-*3*-*phosphate* (*g3p*) genes of *P. ovale* spp., *dihydropoteroate synthase* (*dhps*) of *P. malariae* [43], *dihydrofolate reductase* (*dhfr*) and *MSP*-*1* genes in both species [44–46]. Amplified fragments of DNA were purified, and sequenced directly or cloned into pMD18-T vector (Takara) for sequencing. Sequences were assembled using Lasergene (DNASTAR). The sequences determined in this study were deposited in GenBank with accession numbers KX672017-KX672048.

### Sequence analysis

Sequences were searched in the GenBank by using the BLAST program. Orthologous sequences of *P. malariae* and *P. ovale* spp. were retrieved from the GenBank and PlasmoDB (http://www.plasmodb.org) for phylogenetic analysis. GenBank accession numbers of the genes included in sequence analysis are shown in Additional file 2. Nucleotide and deduced amino acid sequences were aligned by using CLUSTALW. Phylogenetic trees were constructed using the Neighbor-Joining method implemented in MEGA6 [47].

## Results

### Malaria case detection under the PCD and ACD efforts

For malaria surveillance along the China–Myanmar border, PCD was conducted in clinics and hospitals, while ACD and CSS were performed in the surrounding villages and IDP camps near Laiza township of Kachin State, Myanmar. On the Myanmar side, malaria diagnosis in hospitals and clinics was performed primarily by microscopy, and overall 28.3% of patients with febrile illness were diagnosed as having malaria infections and treated (Table 1). In Kachin State, the ACD efforts detected only one malaria case from 77 (1.3%) villagers with fever symptoms. Malaria prevalence in the villages and camps was low; CSS only detected 1.9% of the participants as having asymptomatic *Plasmodium* infections (Table 1). More specifically, malaria prevalence (slide positivity) in the villages was 1.3% (8/638), whereas it was slightly higher (2.9%, 12/411) in the IDP camps. In this border region, *P. vivax* and *P. falciparum* were the predominant parasite species. In Myanmar, among the 437 malaria cases identified by microscopy, *P. vivax*, *P. falciparum* and *P. malariae* infections accounted for 61.1, 30.2 and 0.2%, respectively (Table 1). In addition, 8.5% of these cases were diagnosed as mixed *P. falciparum*/*P. vivax* infections. In the four border counties of Yunnan Province, China, 59.2% of febrile cases suspected for malaria infections were diagnosed by an RDT as malaria cases. Among the 61 positive cases, 68.9, 29.5 and 1.6% were due to *P. vivax*, *P. falciparum* and mixed infections of these two species.

For a more accurate identification of parasite species, nested PCR targeting the parasite SSU rRNA genes was used to analyze the 2701 blood samples. For both PCD and CSS efforts, PCR detected more malaria cases (Table 1). For PCD in Myanmar, microscopy missed 9.8% (45/461) of malaria cases among the febrile patients. Furthermore, PCR detected 13.7% (63/461) of the malaria cases as mixed *P. falciparum/P. vivax* infection as compared to 7.4% (34/461) determined by microscopy. For the CSS efforts, PCR detected 2.9% of participants carrying *Plasmodium* infections, an increase of 1% compared with that of microscopic diagnosis. In Yunnan, PCR also improved the RDT diagnosis sensitivity by 7.8%. In comparison, there were 41 discrepancies between the results of microscopy and PCR (Additional file 3). Six were slide-positive but PCR negative (one *P. falciparum* and five *P. vivax*). Of 26 cases diagnosed as mixed-species infections by microscopy, 22 were *P. falciparum* and 4 were *P. vivax* single infections. In addition, nine slide-positive cases were identified as infections by a different species (Additional file 3).

For the minor *Plasmodium* species, whereas no *P. knowlesi* infections were identified, PCR detected a total of four *P. malariae* and three *P. ovale* cases (Table 1). Of the four *P. malariae* cases, only one was correctly identified, and the remaining three were misdiagnosed as *P. vivax* infections by microscopy. In addition, one *P. malariae* case was diagnosed by the RDT as a *P. vivax* infection. Two *P. ovale* cases were missed by both microscopy and RDT, probably due to low parasitemias, whereas one was misdiagnosed as *P. vivax* infection. Both parasite species had very low prevalence of 1.2% (7/561) in PCR-confirmed positive cases. In total, infections by the two minor *Plasmodium* species only made up 0.26% (7/2701) of all febrile cases at the hospitals and clinics. It is also noteworthy that all *P. malariae* and *P. ovale* cases were from PCD surveys. Most *P. malariae* and *P. ovale* patients presented with fever at the time of examination (>37.5 °C) and had 2–5 days of fever histories (Table 2). These cases with disparate diagnosis results between the original microscopy and PCR were re-examined by an expert microscopist, who confirmed the PCR results.

**Table 2:** Characteristics of the *P. malariae* and *P. ovale* cases

Case number	Age	Sex	Microscopy	RDT	PCR	Axillary temperature	Parasite density^c^	
							Asexual	Gametocytes
M0500214^a^	27	M	–	–	*P. ovale*	40.0	–	–
M0102751^a^	7	M	–	–	*P. ovale*	38.6	–	–
C0100511^b^	33	M	Pv	Pv	*P. ovale*	38.9	800	400
M0N00648^a^	30	M	Pm	ND	*P. malariae*	38.0	880	40
M0N00556^a^	11	F	Pv	ND	*P. malariae*	39.5	2120	4800
M0N00290^a^	29	M	Pv	ND	*P. malariae*	36.0	80	1600
C0400117^b^	22	M	Pv	Pv	*P. malariae*	40.5	400	160

*ND* not done

^a^Cases detected in Myanmar

^b^Cases detected in Yunnan

^**c**^Parasites/μl of blood

### Confirmation of *P. malariae* and *P. ovale* cases

**Fig. 1:**
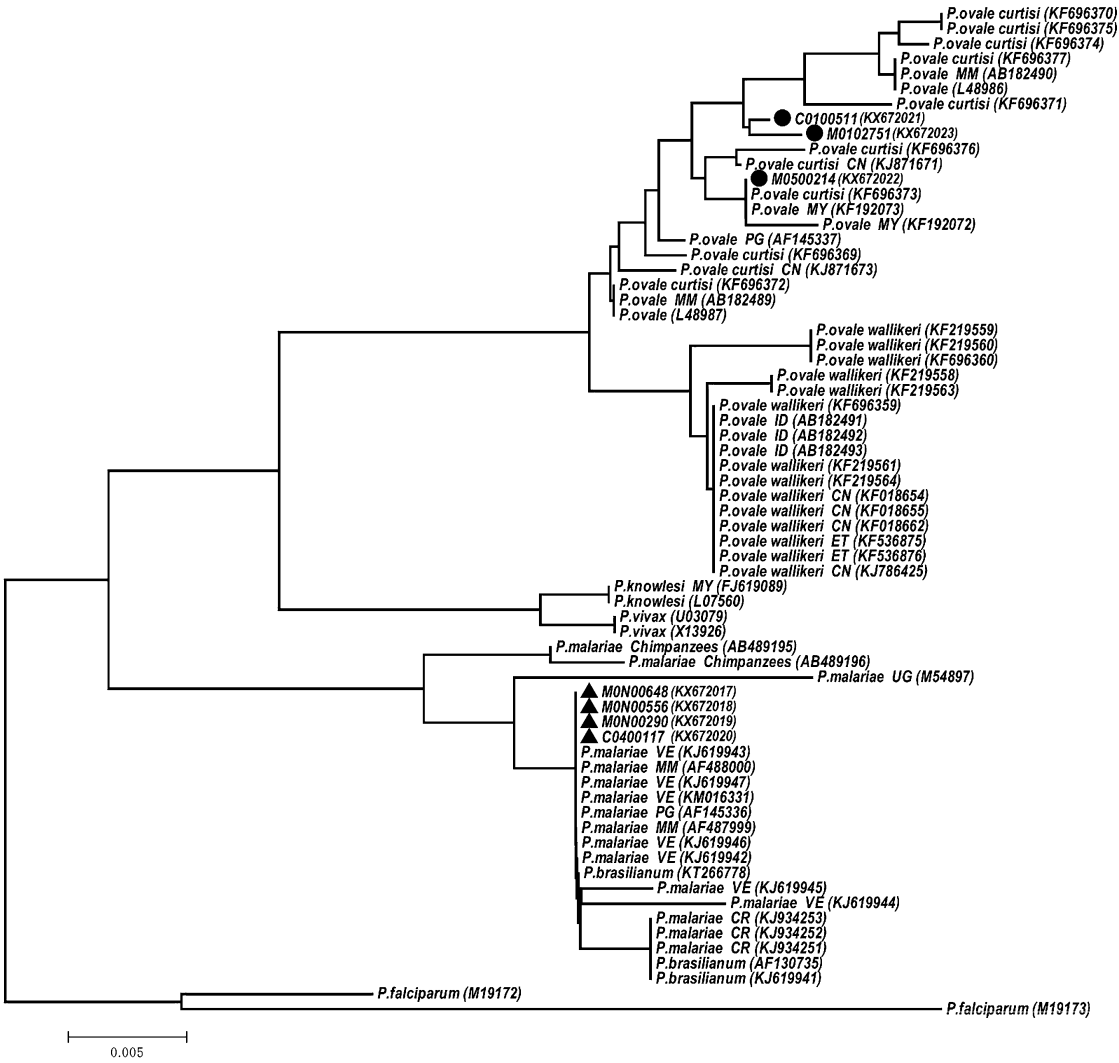
Phylogenetic relationship based on the SSU rRNA sequences from *Plasmodium* species (GenBank accession numbers are in *brackets*)*. Black circles* and *triangles* represent the *P. ovale* and the *P. malariae* isolates respectively from China–Myanmar border area in this study. *CN* China; *CR* Costa Rica; *ET* Ethiopia; *ID* Indonesia; *MM* Myanmar; *MY* Malaysia; *PG* Papua New Guinea; *VE* Venezuela

The PCR products of the SSU rRNA genes of the two minor species from the positive cases were cloned and sequenced. BLAST analysis and alignment of sequences showed that the four *P. malariae* SSU rRNA gene sequences had 99% identities with those from published *P. malariae* sequences, whereas the *P. ovale* SSU rRNA gene sequences from the three isolates were most closely related to those of *P. ovale* spp. with 99% identities. Consistently, phylogenetic analysis showed all four *P. malariae* SSU rRNA gene sequences were grouped together with other *P. malariae* sequences, whereas the three clinical *P. ovale* isolates were clustered with *P. ovale curtisi* (Fig. 1).

### *Plasmodium ovale* subtype characterization

*Plasmodium ovale* spp. are divided into two genetically distinct subspecies, *Poc* and *Pow* [12]. To further confirm that the *P. ovale* isolates identified in this study belong to *Poc*, two mitochondrial genes *Pocytb* (508 bp) and *Pocox1* (861 bp), and one nuclear gene *Pog3p* (359 bp) from the three clinical isolates we amplified, sequenced and compared with the reference sequences (Table 3). It is obvious that the three clinical isolates were more closely related to *Poc* than to *Pow*. Phylogenetic analysis of these three genes all confirmed that the three *P. ovale* isolates belonged to the *Poc* subtype.

**Table 3:**
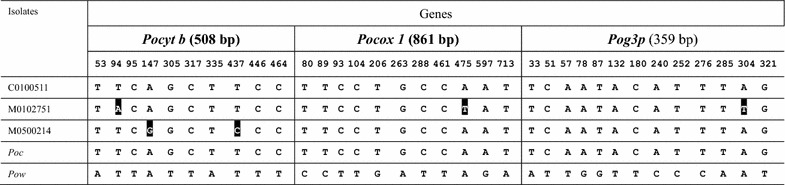
Nucleotide substitutions in *Pocyt b*, *Pocox 1* a*nd Pog3p* compared with the sequences in *Poc* and *Pow*

Numbering was according to the submitted sequence to GenBank. The GenBank access numbers: *Pocyt b*: KX672027 (M0500214), KX672028 (M0102751), KX672029 (C0100511); *Pocox 1*: KX672024 (M0500214), KX672025 (M0102751), KX672026 (C0100511); *Pog3p*: KX672030 (M0500214), KX672031 (M0102751), KX672032 (C0100511). The GenBank access numbers of the reference sequences for the three *Poc* genes are HQ712052, HQ712052 and KP050383, respectively; and for the three *Pow* genes are KP050428, KP050417, and KP050384, respectively. Nucleotides that are different from the type *Poc* sequence are shadowed

### Diversity of the *MSP*-*1* genes

To study the genetic diversity of these two minor *Plasmodium* species in the China–Myanmar border area, four overlapping fragments of the *Pmmsp*-*1* gene and three overlapping fragments of the *Pomsp*-*1* gene were amplified by nested PCR from three (3/4) *P. malariae* isolates (M0N00290, M0N00556 and M0N00648) and two (2/3) *P. ovale* isolates (C0100511 and M0500214), and sequenced. The assembled *PmMSP*-*1* sequences were 5160 bp (M0N00648) and 5150 bp (M0N00290 and M0N00556) in length, whereas the *PoMSP*-*1* sequences were both 5157 bp. Alignment of the *PmMSP*-*1* sequences with the full-length sequence of a Cameroonian isolate MM1A (GenBank #FJ824669) [45] revealed nucleotide and amino acid identities of ~98 and ~97%, respectively. Alignment of the *PmMSP*-*1* sequences from this study with the sequences of 11 Brazilian isolates by fragments showed that fragment 2 sequences corresponding to amino acids 637–858 in the MM1A sequence were the most polymorphic with 87.3–88.9 and 82.3–83.6% identities in the nucleotide and amino acid sequences, respectively (Additional file 4). Fragment 4, corresponding to amino acids 1366–1512 in the MM1A sequence, was absolutely conserved in the *P. malariae* isolates analyzed in this study.

The two *Pomsp*-*1* sequences encoding 1718 amino acids were 100% identical. They were >98% identical in nucleotide sequence with the *Pocmsp*-*1* sequence from the Cameroonian isolates OM1A and OM1B (GenBank #FJ824670 and FJ824671), and ~91% identical with the *Powmsp*-*1* from the Thai isolates (Additional file 5). Comparison of the amino acid sequences showed a similar trend. Compared with the *PoMSP*-*1* gene from the Thai isolate Po-7 (GenBank No. KC137346) [48], there are two synonymous mutations (C421T and T1929C) and one nonsynonymous mutation (C541T) in the isolate C0100511. A 12-nucleotide repeat sequence (GCCGCTACACAA) encoding amino acids AATQ between the position 2814–2911 in the variable domain 5 was found to have repeated three times in the Thai isolate and four times in both C0100511 and M0500214 (Additional file 6). The highest amino acid identity in the other variable domains and interspecies conserved sequences of *PoMSP*-*1* was observed among the M0500214, C0100511 and five *Poc* isolates (Po-4, Po-7, Po-8, Po-9, Po-10) from Thailand.

### The *dhfr*-*ts* genes from *P. malariae* and *P. ovale* isolates

The *dhfr*-*ts* gene from the four *P. malariae* isolates and three *Poc* isolates were amplified and sequenced. The four *P. malariae dhfr*-*ts* genes ranged in size from 1678 to 1779 bp, encoding proteins of 559–592 amino acids and corresponding to amino acids 13–604 of the PmDHFR-TS from the Thai isolate Pm3 (GenBank #EF188271) [44] (Additional file 7). Three of the four isolates from the China–Myanmar border area had a PmDHFR sequence identical to that of the Thai Pm3 isolate, whereas M0N00648 contained two new mutations, L137P and K161R (Table 4; Additional file 7). All four PmDHFR-TS sequences from the China–Myanmar border isolates were wild type at amino acids S49, N50, S58, S114 and I170, which correspond to amino acids C50, N51, C59, S108 and I164 in PfDHFR, respectively. Mutations at these sites are known to confer pyrimethamine resistance [49, 50]. Two of the four PmDHFR sequences also contained amino acids A15 corresponding to A16 in PfDHFR. Of note, the S49 and S58 in PmDHFR-TS were different from the C50 and C59 in the PfDHFR sequence. Mutations N50K, K55E, S58R, S59A, S114N/G and I170M found in other PmDHFR sequences [44, 51] were not observed in the PmDHFR sequences from the China–Myanmar border isolates (Table 4; Additional file 7).

**Table 4:** Polymorphism in partial sequences of DHFR-TS from *P. ovale* and *P. malariae* isolates in the China–Myanmar border area

*P. falciparum*	*A16*	*C50*	*N51*	M55	K56	Y57	F58	*C59*	A60	T62	T63	*S108*	L131	K155	F162	*I164*	Y226	K341	3D7 Reference
*P. falciparum*	V	R	I	**–**	**–**	**–**	**–**	R	**–**	**–**	**–**	*N/T*	**–**	**–**	**–**	L	**–**	**–**	Mutant type [51]
*P. vivax*	*I13*	C49	N50	M54	K55	Y56	F57	S58	S59	*T61*	T62	*S117*	L140	K164	F171	*I173*	Y235	K357	Pv_Sal-1
*P. vivax*	L	**–**	**–**	**–**	**–**	**–**	L/T	R	**–**	M	**–**	N	**–**	**–**	**–**	L	**–**	**–**	Mutant type [51]
*Po. curtisi*	A15	Y49	N50	I54	S55	Y56	F57	S58	S59	T61	T62	S113	L136	K160	F167	I169	Y231	K370	KP050405 [52]
*Po. curtisi*	S	–	–	–	–	–	–	R	–	**–**	**–**	**–**	**–**	**–**	**–**	**–**	**–**	**–**	KP050408 [52]
*Po. wallikeri*	A15	C49	N50	I54	S55	Y56	F57	S58	S59	T61	T62	S113	L136	K160	F167	I169	Y231	K374	KP050406 [52] ]
*Po. wallikeri*	–	–	–	–	–	C	–	–	–	–	–	–	–	–	–	–	–	–	EU266605 [12]
*Po. wallikeri*	–	–	–	–	–	–	L	–	–	–	–	–	–	–	–	–	–	–	KP050409 [52]
C0100511		Y	N	I	S	Y	F	S	S	T	T	S	L	K	F	I	Y	K	China (KX672038)
M0102751		–	–	–	–	–	–	–	–	–	A	C	–	–	–	–	H	E	Myanmar (KX672039)
M0500214		–	–	–	–	–	–	–	–	–	T	C	–	–	–	–	Y	K	Myanmar (KX672037)
*P*. *malariae*	A15	S49	N50	M54	K55	Y56	F57	S58	S59	T61	T62	S114	L137	K161	F168	I170	F232	K355	EF188271, Pm3 [44]
*P*. *malariae*	–	–	K	–	E	–	–	R	A	–	**–**	N/G	–	S	S	M	**–**	**–**	Mutant type [44, 51]
C0400117		S	N	M	K	Y	F	S	S	T	T	S	L	K	F	I	F	K	China (KX672042)
M0N00290		–	–	–	–	–	–	–	–	–	–	–	–	–	–	–	–	–	Myanmar (KX672043)
M0N00556	A	–	–	–	–	–	–	–	–	–	–	–	–	–	–	–	–	–	Myanmar (KX672041)
M0N00648	A	–	–	–	–	–	–	–	–	–	–	–	P	R	–	–	–	–	Myanmar (KX672040)

For *P. falciparum* and *P. vivax dhfr genes*, mutations related to pyrimethamine resistance are indicated by italics. In the *P. ovale* and *P. malariae dhfr genes*, equivalent residues known to be related to pyrimethamine resistance in the *P. falciparum* and *P. vivax dhfr genes* are shown in underlines only.

The three sequences of the *Podhfr*-*ts* gene ranged from 1673 to 1757 bp and encode proteins of 557–585 amino acids, corresponding to amino acids 24–608 of the PocDHFR-TS from the Indian isolate Po2003 (GenBank #KP050405) [52] (Additional file 7). Sequence polymorphisms were found at positions A62, C113, H231 and E370 in PocDHFR-TS from the isolate M0102751, while isolate M0500214 only contained C113 (Table 4; Additional file 7). Similarly, Y49 and S58 in PocDHFR-TS were different from C50 and C59 in the PfDHFR-TS sequence. Also, C113 in PocDHFR-TS from the isolate M0102751 and M0500214 was different from S108 in the PfDHFR-TS **(**Table 4).

### Sequencing analysis of the *dhps* genes from *P. malariae*

The *Pmdhps* gene was successfully amplified from the four *P. malariae* isolates. The partial *Pmdhps* gene was 962 bp in length and encoded 320 amino acids, corresponding to amino acids 347–659 in PfDHPS. The four PmDHPS sequences from the China–Myanmar border area isolates were identical to the PmDHPS haplotype 1 reported earlier [43] (Additional file 8). The six amino acids in PmDHPS equivalent to the residues associated with sulfadoxine resistance in PfDHPS (S436, K540, A581, I588 and A613) were all wild type except the mutant A437G.

## Discussion

Accurate knowledge of the malaria epidemiology is essential for guiding control and elimination efforts. This study employed both ACD and PCD along the international borders between China and Myanmar, and used a molecular detection method in order to more accurately determine the infection prevalence among febrile patients and asymptomatic residents, and parasite species compositions. In this border region, malaria is seasonal with all four human malaria parasites. While *P. vivax* has become the predominant parasite species followed by *P. falciparum* [16, 53], the two minor parasite species *P. malariae* and *P. ovale* spp. were detected at very low frequencies in patients presenting in hospitals and clinics with acute febrile illness. It is important to note that three of the four *P. malariae* cases and one *P. ovale* case were misidentified as *P. vivax* infections by microscopy and treated with the chloroquine/primaquine combination, whereas two *P. ovale* cases were missed by microscopy and thus were not treated with anti-malarial drugs. Furthermore, PCR also identified more mixed-species infections, which may not have been treated properly depending on the results of the initial diagnosis. Thus, more sensitive diagnosis may well be needed for “border malaria” in order to completely eliminate malaria transmission.

Published data to date showed that the *dhfr* genes in *Poc* and *Pow* were highly conserved in codons potentially mediating pyrimethamine resistance, although isolates carrying double A15S and S58R mutations were observed in imported cases in Singapore [12, 52]. The four *P. malariae* cases and three *P. ovale* cases detected in this study did not show mutations at equivalent residues responsible for antifolate resistance in other *Plasmodium*, while two new mutations at positions L137P and K161R were observed in one *P. malariae* isolate and four new mutations were observed at positions T62A, S113C, Y231H and K370E in *Podhfr*. It is noteworthy that the S113C mutation in the *Pocdhfr* corresponds to amino acid S108 in *Pfdhfr*, the primary position associated with pyrimethamine resistance [50, 54], but it is not known whether S113C mutation also confers pyrimethamine resistance in *Poc*. Similarly, *Pmdhps* gene also showed limited genetic diversity and all four isolates from the China–Myanmar border were identical to the PmDHPS haplotype 1, which was most prevalent in Asian countries [43].

MSP-1 as a predominant merozoite surface antigen is present in all examined *Plasmodium* species. *P. falciparum* and *P. vivax MSP*-*1* genes display extensive genetic diversity, which is often used as a molecular marker for population studies. To date, limited studies on *PmMSP*-*1* among Brazilian isolates and *PocMSP*-*1* on Thai isolates detected low-level sequence diversity with most of variations located at interspecies variable domains of this gene [46, 48]. Phylogenetic analysis of *PmMSP*-*1* using the maximum likelihood method placed the China–Myanmar border isolates separated from the Brazilian isolates, indicating divergent parasite populations. Furthermore, the *PocMSP*-*1* sequences from the China–Myanmar border isolates were highly conserved and also displayed a high level of similarity with the *PocMSP*-*1* from the Thai isolates. Apparently, future studies with larger populations of the two minor parasite species are warranted.

## Conclusions

Malaria epidemiology at the China–Myanmar border has shifted to *P. vivax* predominance, while the two minor parasite species *P. malariae* and *P. ovale* spp. also occurred at very low prevalence. However, these minor species were most often misidentified or missed, highlighting a potential problem in malaria treatment. Molecular studies identified the *P. ovale* spp. as the subspecies *Poc*. Characterization of two antifolate genes *dhfr* and *dhps* revealed significant conservation at most positions possibly conferring antifolate resistance in *P. falciparum* and *P. vivax*, suggesting reduced impacts of antifolate selection on these two minor parasite species. Despite this, the S113C mutation, corresponding to S108 position in *Pfdhfr*, was first reported here in two Poc isolates from the China–Myanmar border area. Further analysis of the *MSP*-*1* gene also revealed much lower levels of genetic diversity than their orthologs in *P. falciparum* and *P. vivax* populations, possibly due to the persistent low prevalence of these minor species in the GMS.

## Additional files

**Additional file 1.** PCR primer sequences and reaction conditions.

**Additional file 2.** GenBank accession number of the orthologous sequences.

**Additional file 3.** Discrepancies in diagnosis between microscopy and PCR.

**Additional file 4.** Sequence identity between the *PmMSP*-*1* sequences.

**Additional file 5.** Sequence identity between the *PoMSP*-*1* sequences.

**Additional file 6.** Alignment of PoMSP1 sequences.

**Additional file 7.** Sequence alignment of PmDHFR-TS (A) and PoDHFR-TS (B).

**Additional file 8.** Sequence alignment of PmDHPS.

## Abbreviations

GMS: Greater Mekong Subregion
PCD: passive case detection
CSS: cross-sectional surveys
ACD: active case detection
IDP: internally displaced people
RDT: rapid diagnostic tests
*msp-1*: *merozoite surface protein-1*
*cytb*: *cytochrome b*
*cox1*: *cytochrome oxidase subunit 1*
*g3p*: *glyceraldehyde-3-phosphate*
*dhps*: *dihydropoteroate synthase*
*dhfr*: *dihydrofolate reductase*
