# Supplementary material for: Plasmodium malariae and Plasmodium ovale infections in the China–Myanmar border area
Source: Malar J. 2016 Nov 15;15:557. doi: 10.1186/s12936-016-1605-y (PMC5111346; doi:10.1186/s12936-016-1605-y)
Supplement: Supplementary file 1 — Additional file 1. PCR primer sequences and reaction conditions. [file 12936_2016_1605_MOESM1_ESM.pdf]

Additional file 1. PCR primer sequences and reaction conditions

Gene	Primer name	Primer sequence	PCR conditions	PCR product size (bp)	Ref. sequences (Genbank No.)
Pocyt b	Pocytb-F1<br>Pocytb-R1<br>Pocytb-F2<br>Pocytb-R2	GATATATGCATGCAACTGGTGCA<br>GAACACATAAACCATATAATTGGAA<br>CAATTCCAGCCTTAGTTATATGGT<br>GAATATTCTCTAGCACCAAATGCCA	The first PCR: 94 ^{o}C for 5 min; 94 ^{o}C for 1 min, 50 ^{o}C for 1 min, 70 ^{o}C for 2 min, 30 cycles; 70 ^{o}C 10 min.<br>The second PCR: 94 ^{o}C for 5 min; 94 ^{o}C for 50 sec, 55 ^{o}C for 50 sec, 70 ^{o}C for 2 min, 35 cycles; 70 ^{o}C 10 min.	508	FJ409567
Pocox1	Pocox1-F1<br>Pocox1-R1<br>Pocox1-F2<br>Pocox1-R2	ATGCCTGGATTATTTGGAGGA<br>TCCTATAATGGCACCAATAG<br>TACCTATTTTATGTGGATCACC<br>CATGTAATGCTATATCAATAGCT	The first PCR: 94 ^{o}C for 5 min; 94 ^{o}C for 1 min, 50 ^{o}C for 1 min, 70 ^{o}C for 2 min, 30 cycles; 70 ^{o}C 10 min.<br>The second PCR: 94 ^{o}C for 5 min; 94 ^{o}C for 50 sec, 55 ^{o}C for 50 sec, 70 ^{o}C for 2 min, 35 cycles; 70 ^{o}C 10 min.	861	FJ409571
Pog3p	Pog3p-F1<br>Pog3p-R1<br>Pog3p-F2<br>Pog3p-R2	GCAGGAATAATATTTTGTAAGGCACA<br>GCATGTTCACCGTTGGCGATAAGAA<br>CCTTTAGATGGTCCATCAACTACTA<br>GTACACAGCGAGAAGGATCCAGCT	The first PCR: 94 ^{o}C for 5 min; 94 ^{o}C for 1 min, 50 ^{o}C for 1 min, 70 ^{o}C for 2 min, 30 cycles; 70 ^{o}C 10 min.<br>The second PCR: 94 ^{o}C for 5 min; 94 ^{o}C for 50 sec, 55 ^{o}C for 50 sec, 70 ^{o}C for 2 min, 35 cycles; 70 ^{o}C 10 min.	359	KP050383
Pomsp1	PoMSP1-outerF1<br>PoMSP1-outerR1<br>PoMSP1-innerF1<br>PoMSP1-innerR1<br>PoMSP1-innerF2<br>PoMSP1-innerR2<br>PoMSP1-innerF3<br>PoMSP1-innerR3	ATGAAGGTGTTCGTATTTGCGCTC<br>GGATCAATAAGACTGCCAATAAGA<br>GTTCGTATTTGCGCTCTCTTTCATT<br>CTACATTAGATAATGGTAAGGTAGA<br>CTACCTTACCATTATCTAATGTAGTA<br>CACAGTTTCCACTTTTGGAAGCAA<br>CTTCCAAAAGTGGAAACTGTGGCT<br>GCCAATAAGAAAGATAAGCTAAGGA	The first PCR: 95 ^{o}C for 5 min; 95 ^{o}C for 30 sec, 62 ^{o}C for 5 min, 30 cycles; 72 ^{o}C 10 min.<br>The second PCR: 95 ^{o}C for 5 min; 95 ^{o}C for 40 sec; 62 ^{o}C for 1 min, 72 ^{o}C for 150 sec, 35 cycles; 72 ^{o}C 10 min.	5157<br>1814<br>2033<br>1358	FJ824670
Pmmsp1	PmMSP1-outerF<br>PmMSP1-outerR<br>PmMSP1-innerF1<br>PmMSP1-innerR1<br>PmMSP1-innerF2<br>PmMSP1-innerR2<br>PmMSP1-innerF3<br>PmMSP1-innerR3<br>PmMSP1-innerF4<br>PmMSP1-innerR4	GTCACTGTGAAACAAATGAAGAT<br>CCTCTTGCTCAGTCTTAATCCTT<br>GGAAAACTGGAGGAACTAGTTGT<br>GTCTTCTAAATACATAAGTTGCT<br>CTTGATAATAGCATTACAACAGAGA<br>GCTCACACTGTCATACATTGAA<br>GGAATCGTTGTTCCGACATTGT<br>GCATTATCCGGTTGCTCTTCT<br>CCTACTTATCTGGTGGATTACAT<br>CTCATTTTATGTTCATTCGTATCCT	The first PCR: 95 ^{o}C for 5 min; 95 ^{o}C for 40 sec, 55 ^{o}C for 1 min, 72 ^{o}C for 5 min, 30 cycles; 72 ^{o}C 10 min.<br>The second PCR: 95 ^{o}C for 5 min; 95 ^{o}C for 40 sec, 60 ^{o}C for 1 min, 72 ^{o}C for 2 min, 35 cycles; 72 ^{o}C 10 min.	5150-5160<br>1497<br>1293<br>1484<br>1499-1509	FJ824669
Podhfr	Podhfr-outerF<br>Podhfr-outerR<br>Podhfr-innerF<br>Podhfr-innerR	CGCGATATGCGCGTGCTGCAAA<br>CGTGGTGGACATAATTCTCTAT<br>GAGATTGGAAGAAGAGTGAATCGT<br>TTCCGGGTTTAGTTTAAGCGTG	The first PCR: 94 ^{o}C for 5 min; 94 ^{o}C for 40 s; 55 ^{o}C for 1 min; 72 ^{o}C for 120 sec, 30 cycles; 72 ^{o}C 10 min<br>The second PCR: 94 ^{o}C for 5 min; 94 ^{o}C for 40 sec, 60 ^{o}C for 1 min, 72 ^{o}C for 120 sec, 35 cycles; 72 ^{o}C 10 min.	1673-1757	EU266605
Pmdhfr	Pmdhfr-outerF<br>Pmdhfr-outerR<br>Pmdhfr-innerF<br>Pmdhfr-innerR	ATGGAGGAAGTCTCAGACGTATTCGAT<br>TAGGCGGCCATATCCATTGTTA<br>CATCTGCGCTTGCTGTAAAGTGCCA<br>GTAAAATCCGATATTGTAAAGTCTTCA	The first PCR: 94 ^{o}C for 5 min; 94 ^{o}C for 40 s; 55 ^{o}C for 1 min; 72 ^{o}C for 120 sec, 30 cycles; 72 ^{o}C 10 min<br>The second PCR: 94 ^{o}C for 5 min; 94 ^{o}C for 40 sec, 60 ^{o}C for 1 min, 72 ^{o}C for 120 sec, 35 cycles; 72 ^{o}C 10 min.	1685-1779	EF188271
Pmdhps	Pmdhps-outerF<br>Pmdhps-outerR<br>Pmdhps-innerF<br>Pmdhps-innerR;	ATACCTAATTATAAACATATTG<br>ACCACATATATTTTTCTGATAT<br>GATACGATAAACACATTGTATT<br>TGGTCTTTATCCTTGCGCAT	The first PCR: 94 ^{o}C for 5 min; 94 ^{o}C for 30 sec, 45 ^{o}C for 45 sec, 70 ^{o}C for 120 sec, 30 cycles; 72 ^{o}C 10 min.<br>The second PCR: 94 ^{o}C for 5 min; 94 ^{o}C for 30 sec, 52 ^{o}C for 45 sec, 72 ^{o}C for 120 sec, 35 cycles; 72 ^{o}C 10 min.	962	KJ400020
