# Supplementary material for: Plasmodium malariae and Plasmodium ovale infections in the China–Myanmar border area
Source: Malar J. 2016 Nov 15;15:557. doi: 10.1186/s12936-016-1605-y (PMC5111346; doi:10.1186/s12936-016-1605-y)
Supplement: Supplementary file 2 — Additional file 2. GenBank accession number of the orthologous sequences. [file 12936_2016_1605_MOESM2_ESM.pdf]

Additional file 2. GenBank accession number of the orthologous sequences

Genes	**GenBank accession No. (Isolate)**
P. falciparum SSU rRNA	M19172 (in asexual parasites) M19173 (not in asexual parasites)
P. vivax SSU rRNA	X13926 U03079 (Sal 1)
P. malariae SSU rRNA	AF487999 (type 1) AF488000 (type 2) KJ619942 (KOY13) AF145336
	AB489195 (Takaboh) AB489196 (Oumu) KJ619943 (OCA24) KM016331
	M54897, KJ619943-KJ619947 (VEN01-VEN04), KJ934251-KJ934253 (CR1-CR4)
P. brasilianum SSU rRNA	KJ619941 (HA12) KT266778 (Peruvian) AF130735
P. knowlesi SSU rRNA	FJ619089 (LT20-C4) L07560
P. ovale spp. SSU rRNA	KF536875 (*Pow*: PoW4) KF018654 (ZBP-HN clone RH1)
	KF536876 (*Pow*: PoW6) KF018655 (ZBP-HN clone RH2)
	KF192072 (MAL-1) KF018662 (ZBP-HN clone BZ-5H2)
	KF192073 (MAL-2) KF219563 (*Pow* clone 5SH5)
	KF696359 (*Pow* clone GC-1) KF219564 (*Pow* clone 5SH8)
	KF696360 (*Pow* clone GC-2) KF696369-KF696377 (*Poc* clone DC-1 to DC-10)
	KJ786425 (*Pow* FJ11) KF219558-KF219561 (*Pow* clone RSH1, 2, 8, 10)
	KJ871671 (*Poc*, FJX4) AB182489-AB182490 (Classic type 1-2)
	KJ871673(*Poc*, FJX22) AB182491-AB182493 (Variant type 1-3)
	AF145337 L48987 L48986 (clone 26)
Pocytb SSU rRNA	KP050427 (Po2003) GU723537(UKMRL08) HQ712052
	KP050428 (Po2006) G U723534(UKMRL34) FJ409567
Pocox1 SSU rRNA	KP050416 (Po2003) JF894415 (PoC15) HQ712052 FJ409571
	KP050417 (Po2006) JF894419 (PoW1) (*Poc*)
Pog3p SSU rRNA	KP050383 (Po2003) GU723549 (UKMRL01)
	KP050384 (Po2006) GU723571(UKMRL02)
P. malariae MSP-1	FJ824669 (Cameroon isolate MMA1)
	Brazilian isolates:
	KR072269-KR072279 (fragment 1) JX045643-JX045645 (fragment 2)
	KR072215-KR072220 (fragment 2) KR072254-KR072263 (fragment 3)
	KR072239-KR072248 (fragment 4) KR072223 -KR072233 (fragment 5)
P. ovale spp. MSP-1	KC137340-KC137349 (Thailand isolates PO-1 to PO-10)
	FJ824670 (Cameroon OMIA) FJ824671 (Cameroon OM1B)
P. falciparum dhfr-ts	XM_001351443 (3D7) KI926308 (Tanzania 2000708)
P. vivax dhfr-ts	XM_001615032 (SAI-1)
P. malariae dhfr-ts	AY846633 (Thai isolate) EF188271 (Pm3) EF188272 (Te) EF188273 (Tu)
	AY846634 (Laos isolate) EF198109 (Ch) EF198110 (N) EF198111 (O)
P. ovale spp. dhfr-ts	KP050405 (Po2003) KP050411 (Po2013-1) EU266605 (Po4)
	KP050406 (Po2006) KP050412 (Po2013-2) EU266606 (Po3)
	EU266608 (17w) EU266609 (Po18) EU266607 (Po4/2)
	EU266610 (Po19) KP050407-KP050410 (Po2012-1 to Po2012-4)
P. falciparum dhps	XM_001349382 (3D7) KI926388 (Tanzania 2000708)
P. vivax dhps	XM_001617159 (SaI-1)
P. malariae dhps	KJ400020 (PmS) KJ400022 (YA) KJ400023 (Av427) KJ400024 (Pm5)
	KJ400025(AV1373) KJ400026 (L138) KJ400027 (T4) KJ400028 (W13)
