# Supplementary material for: Plasmodium malariae and Plasmodium ovale infections in the China–Myanmar border area
Source: Malar J. 2016 Nov 15;15:557. doi: 10.1186/s12936-016-1605-y (PMC5111346; doi:10.1186/s12936-016-1605-y)
Supplement: Supplementary file 3 — Additional file 3. Discrepancies in diagnosis between microscopy and PCR. [file 12936_2016_1605_MOESM3_ESM.pdf]

**Additional file 3 Discrepancies in diagnosis between microscopy and PCR**

| **Microscopy** | Pv | Pf | Pm | Pv | Pf | Pv | Mixed | |
|---|---|---|---|---|---|---|---|---|
| **PCR** | Pf | Pv | Pv | Pm | - | - | Pf | Pv |
| **Case Number** | 3 | 4 | 1 | 1 | 1 | 5 | 22 | 4 |
