# Supplementary material for: Plasmodium malariae and Plasmodium ovale infections in the China–Myanmar border area
Source: Malar J. 2016 Nov 15;15:557. doi: 10.1186/s12936-016-1605-y (PMC5111346; doi:10.1186/s12936-016-1605-y)
Supplement: Supplementary file 4 — Additional file 4. Sequence identity between the PmMSP-1 sequences. [file 12936_2016_1605_MOESM4_ESM.pdf]

Additional file 4. Sequence identity between the *PmMSP-1* sequences

A. Nucleotide identity

	Cameroonian isolate	Brazilian isolates				
	MM1A 5256 bp	fragment 1 533 bp	Fragment 2 657-675 bp	fragment 3 656-680 bp	fragment 4 443 bp	fragment 5 631 bp
M0N00290	98.29	96.62-97.56	87.42-88.89	99.54-99.85	99.77	99.37-99.68
M0N00556	98.22	96.62-97.56	87.26-88.72	99.39-99.71	99.77	99.37-99.68
M0N00648	98.30	96.62-97.56	87.42-88.89	99.54-99.85	99.77	99.37-99.68

B. Amino acid identity

	Cameroonian isolate	Brazilian isolates				
	MM1A 1751 aa	fragment 1 177 aa	fragment 2 219-225 aa	fragment 3 218-226 aa	fragment 4 147 aa	fragment 5 210 aa
M0N00290	97.06	90.96-93.79	82.27-83.58	98.62-99.56	100	98.10-99.05
M0N00556	97.00	90.96-93.79	82.27-83.58	98.62-99.56	100	98.10-99.05
M0N00648	97.07	90.96-93.79	82.27-83.58	98.62-99.56	100	98.10-99.05
