# Supplementary material for: Plasmodium malariae and Plasmodium ovale infections in the China–Myanmar border area
Source: Malar J. 2016 Nov 15;15:557. doi: 10.1186/s12936-016-1605-y (PMC5111346; doi:10.1186/s12936-016-1605-y)
Supplement: Supplementary file 5 — Additional file 5. Sequence identity between the PoMSP-1 sequences. [file 12936_2016_1605_MOESM5_ESM.pdf]

Additional file 5. Sequence identity between the *PoMSP-1* sequences

A. **Nucleotide identity**

	Identities (%)			
	Cameroon isolates		Thailand isolates	
	OM1A<br>5256 bp	OM1B<br>5159 bp	*P. ovale curtisi*<br>5181bp	*P. ovale wallikeri*<br>5016-5043bp
C0100511	98.42	99.22	99.65-99.94	91.00-91.17
M0500214	98.48	99.28	99.71-100	91.02-91.19

B. Amino acid identity

	Identities %			
	Cameroon isolates		Thailand isolates	
	OM1A<br>1730aa	OM1B<br>1718aa	*P. ovale curtisi*<br>1727aa	*P. ovale wallikeri*<br>1672-1681aa
C0100511	96.71	98.18	99.30-99.94	86.02-86.37
M0500214	96.77	98..24	99.36-100	86.08-86.43
