# Supplementary material for: Plasmodium malariae and Plasmodium ovale infections in the China–Myanmar border area
Source: Malar J. 2016 Nov 15;15:557. doi: 10.1186/s12936-016-1605-y (PMC5111346; doi:10.1186/s12936-016-1605-y)
Supplement: Supplementary file 6 — Additional file 6. Alignment of PoMSP1 sequences. [file 12936_2016_1605_MOESM6_ESM.pdf]

Additional file 6. Alignment of PoMSP1 sequences.

(A) Nucleotide substitutions and the 12-nucleotide repeat region of *PoMSP-1* (numbering based on the sequence of the *PoMSP-1* from the Thai isolate Po-7). ***GenBank accession numbers: KX672045 (C0100511), KX672044 (M0500214); Cameroon (CM) isolates: FJ824670 and FJ824671; Thailand (TH) isolates: KC137340-KC137341, KC137343-KC137346 and KC137349.

A.

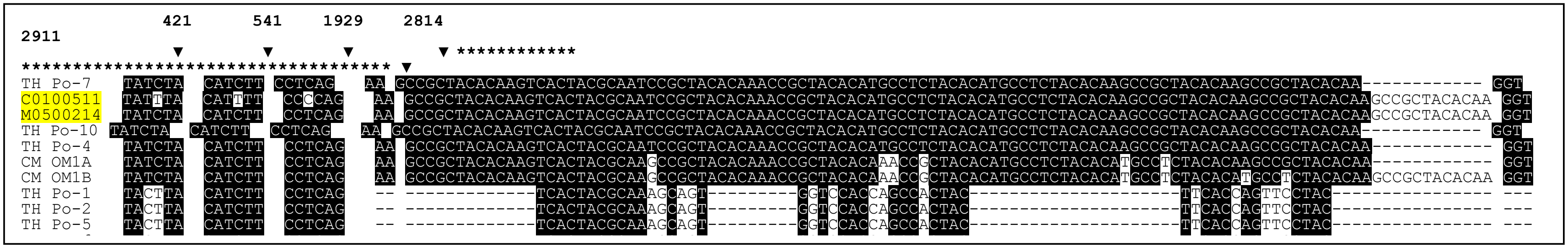

(B) Amino acid polymorphisms in the variable domains of PoMSP-1. Protein sequence numbering was based on the sequence of the PoMSP-1 from the Thai isolate Po-7. *GenBank accession numbers: KX672045 (C0100511), KX672044 (M0500214); Cameroon (CM) isolates: FJ824670 and FJ824671; Thailand (TH) isolates: KC137340-KC137341, KC137343-KC137346 and KC137349.

B.

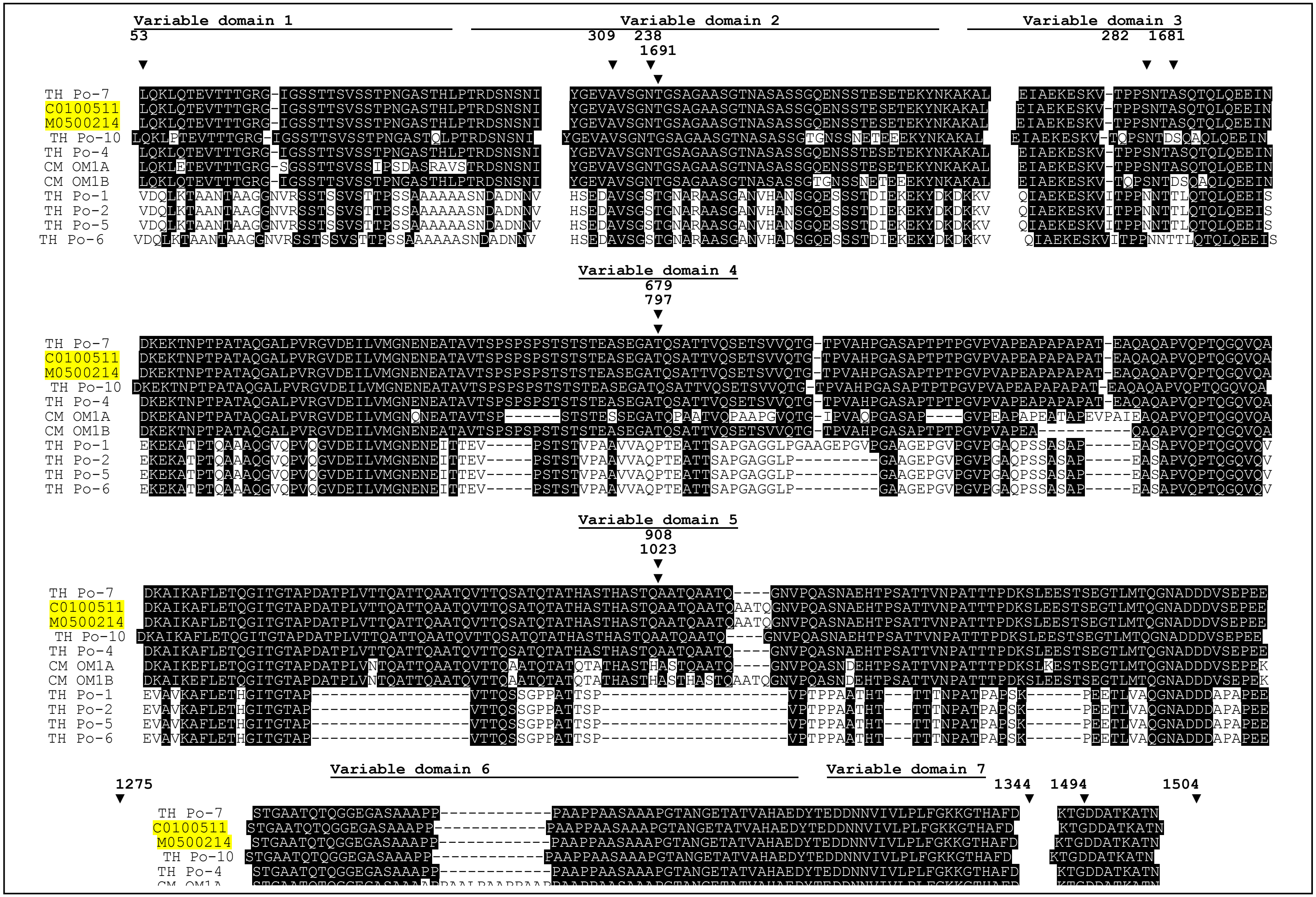
Variable domain 1
Variable domain 2
Variable domain 3
Variable domain 4
Variable domain 5
Variable domain 6
Variable domain 7
53
309
238
1691
282
1681
679
797
908
1023
1275
1344
1494
1504
TH Po-7
C0100511
M0500214
TH Po-10
TH Po-4
CM OM1A
CM OM1B
TH Po-1
TH Po-2
TH Po-5
TH Po-6
