# Supplementary material for: Plasmodium malariae and Plasmodium ovale infections in the China–Myanmar border area
Source: Malar J. 2016 Nov 15;15:557. doi: 10.1186/s12936-016-1605-y (PMC5111346; doi:10.1186/s12936-016-1605-y)
Supplement: Supplementary file 7 — Additional file 7. Sequence alignment of PmDHFR-TS (A) and PoDHFR-TS (B). [file 12936_2016_1605_MOESM7_ESM.pdf]

Additional file 7. Alignment of amino acid sequences of PmDHFR-TS (A) and PoDHFR-TS (B) from the China-Myanmar border area and other countries

A.

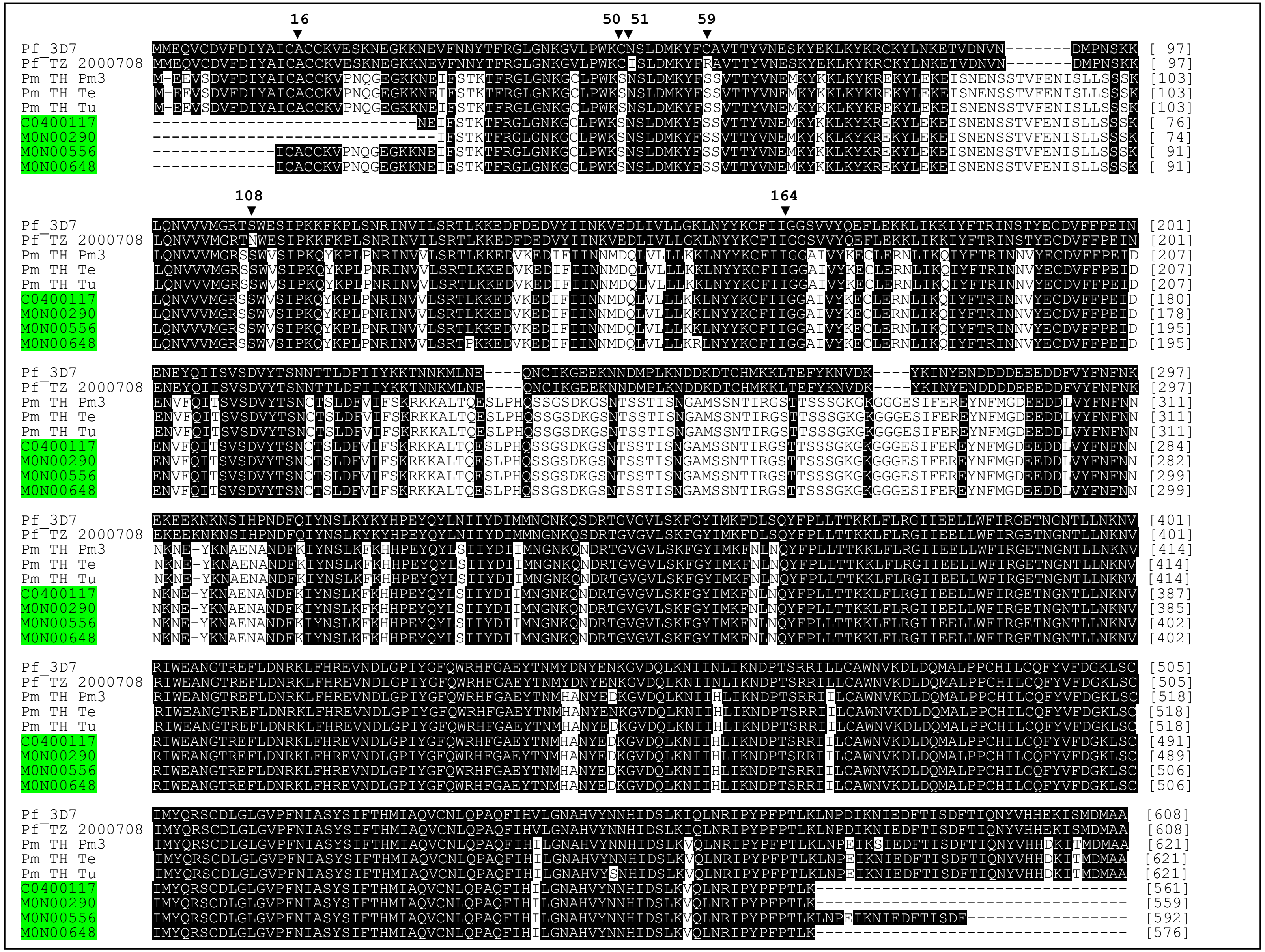

*** GenBank accession numbers:** Isolates of China-Myanmar border: KX672033 (C0400117), KX672034 (M0N00556), KX672035 (M0N00648), KX672036 (M0N00290); Pf: XM_001351443 and KI926308 (Tanzania, TZ 2000708); Thailand (TH) isolates: EF188271 (Pm3), EF188272 (Te) and EF188273 (Tu).

B.

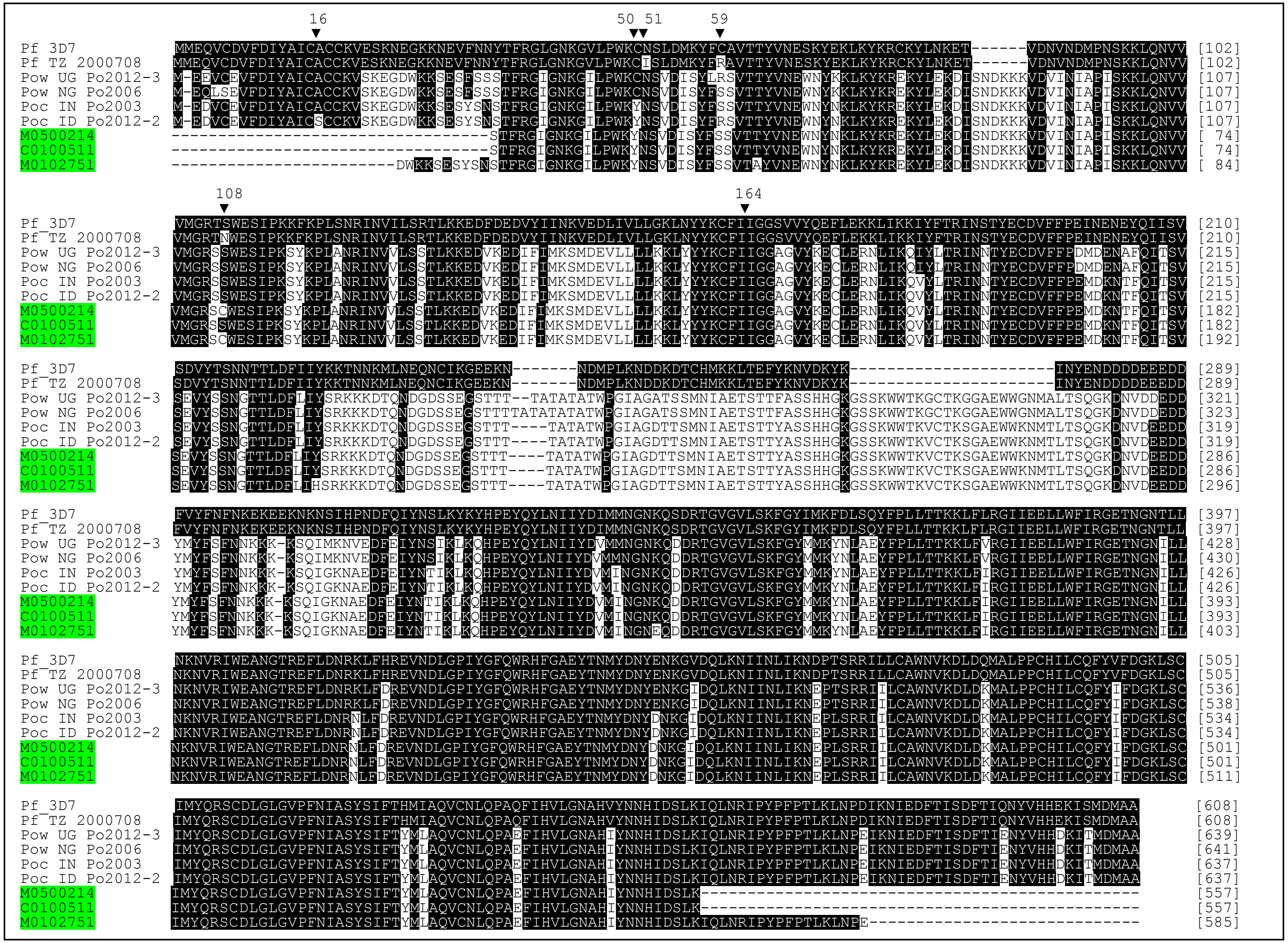

*** GenBank accession numbers:** KX672037 (M0500214), KX672038 (C0100511), KX672039 (M0102751); KI926308 (Tanzania, TZ 2000708); Indonesia (ID): KP050408; India (IN): KP050405; Nigeria (NG): KP050406; Uganda (UG): KP050409.
