# Supplementary material for: Plasmodium malariae and Plasmodium ovale infections in the China–Myanmar border area
Source: Malar J. 2016 Nov 15;15:557. doi: 10.1186/s12936-016-1605-y (PMC5111346; doi:10.1186/s12936-016-1605-y)
Supplement: Supplementary file 8 — Additional file 8. Sequence alignment of PmDHPS. [file 12936_2016_1605_MOESM8_ESM.pdf]

## **Additional file 8.** Sequence alignment of PmDHPS from the China-Myanmar border area and other regions

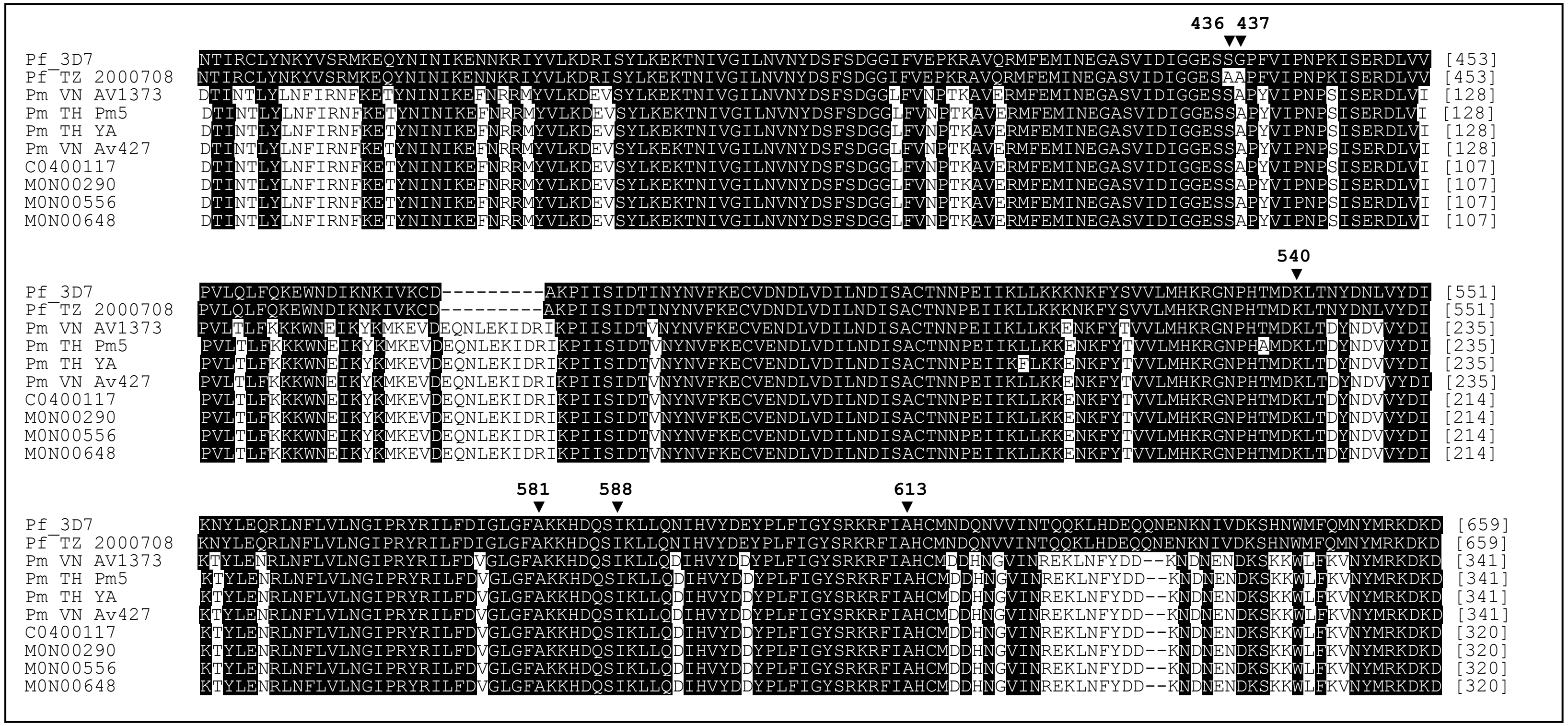


*** GenBank accession numbers:** Isolates of China-Myanmar border: KX672040 (M0N00648), KX672041 (M0N00556), KX672042 (C0400117), KX672043 (M0N00290); Pf: XM_001349382 and KI926388 (Tanzania, TZ 2000708); Thailand (TH) isolates: KJ400022 (haplotype 3) and KJ400024 (haplotype 2). Viet Nam (VN) isolates: KJ400023 (haplotype 4) and KJ400025 (haplotype 1).

Note that Pf 3D7 carries the mutant type A437G.
